# Pathogenic mechanisms of *RPGR* mutations in X-linked retinitis pigmentosa: integrating clinical pedigree and single-cell transcriptomics

**DOI:** 10.3389/fgene.2026.1814462

**Published:** 2026-06-02

**Authors:** XinRong Wang, YuYang Bai, XiaoYang Zuo, XiaoYin Lei, Xue Wang, Gang Zou

**Affiliations:** 1 Third Clinical Medical College, Ningxia Medical University, Yinchuan, Yinchuan, China; 2 Department of Ophthalmology, Ningxia Eye Hospital, People’s Hospital of Ningxia Hui Autonomous Region, Third Clinical School of Medicine, Ningxia Medical University, Yinchuan, China

**Keywords:** ciliary transport, photoreceptor differentiation, *RPGR*, single-cell RNA sequencing, X-linked retinitis pigmentosa

## Abstract

**Purpose:**

This study aims to identify pathogenic retinitis pigmentosa G*TPase regulator* (*RPGR*) mutations in a Chinese pedigree with X-linked retinitis pigmentosa (XLRP) and elucidate the cellular and molecular mechanisms underlying *RPGR*-associated photoreceptor degeneration through the integrated analysis of clinical data and single-cell transcriptomics.

**Methods:**

A three-generation Chinese XLRP pedigree was enrolled for comprehensive ophthalmic examinations, including BCVA, OCT, FAF, and ERG. Whole-exome sequencing was performed on the proband to identify the pathogenic variants, followed by Sanger sequencing for validation in family members. To analyze the downstream molecular mechanisms, we analyzed a public single-cell RNA sequencing dataset (SRP535874) of *RPGR* mutant retinal organoids across four developmental time-points (D40–D200). Bioinformatics analyses included cell clustering, differential expression analysis, GO/KEGG enrichment, protein–protein interaction (PPI) network construction, and pseudotime trajectory analysis.

**Results:**

A hemizygous frameshift mutation (c.2476_2477del; p.R826Gfs*8) in the ORF15 region of *RPGR* was identified in the proband and confirmed in his two sons by Sanger sequencing. Clinical examinations revealed severe retinal degeneration in the affected male, intermediate phenotype in female carriers, and early-stage changes in the young affected male. Single-cell transcriptomic analysis of *RPGR* mutant retinal organoids revealed a paradoxical increase in photoreceptor transcriptional activity at late developmental stages (D150 and D200) despite the loss of the outer retinal structure in the patients, which may reflect aberrant differentiation and impaired functional maturation of photoreceptor precursors. Differential expression analysis showed upregulation of the stress-response genes and downregulation of phototransduction and ciliary transport genes. GO and KEGG enrichment analyses implicated disrupted ribosome biogenesis, RNA metabolism, ubiquitin-mediated proteolysis, and neurodegenerative disease pathways. PPI network analysis indicated decoupling of the core “ciliary transport–phototransduction axis” and activation of a coordinated stress-response module. Pseudotime trajectory analysis showed arrested photoreceptor differentiation at an intermediate stage, preventing the progression to functional maturity.

**Conclusion:**

We identify a previously reported but extremely rare *RPGR* ORF15 frameshift mutation (c.2476_2477del; p.R826Gfs*8) in a Chinese XLRP pedigree. Single-cell transcriptomic analysis indicates that *RPGR* loss-of-function mutation may disrupt the ciliary transport–phototransduction axis, activate stress responses, and block photoreceptor differentiation. These findings expand the *RPGR* mutation spectrum, provide mechanistic insights into XLRP pathogenesis, and have implications for genetic counseling and targeted therapy.

## 1 Introduction

Retinitis pigmentosa (RP) encompasses a cluster of conditions characterized by remarkable clinical and genetic heterogeneity, marked by the gradual degeneration of photoreceptor cells that ultimately culminates in irreversible visual impairment (Karuntu et al., 2025). Among these variants, X-linked retinitis pigmentosa (XLRP), while relatively uncommon, ranks among the most severe subtypes of RP. It manifests in early childhood and constitutes a major cause of blindness in pediatric and adolescent populations (Awadh Hashe et al., 2023). Among the well-characterized pathogenic genes linked to XLRP, mutations in the retinitis pigmentosa G*TPase regulator* (*RPGR*) gene are predominant, accounting for roughly 70%–80% of XLRP cases (Tee et al., 2016). The RPGR protein is localized to the connecting cilium of the photoreceptors, where it interacts with proteins such as *RPGRIP1* to form a “gating” complex at the ciliary transition zone. This complex modulates the selective transport of proteins to the outer segments of photoreceptors; loss of the *RPGR* function disrupts this gating mechanism, leading to deficits in outer-segment protein transport, compromised outer-segment renewal, and eventual photoreceptor degeneration (Megaw et al., 2015). Nevertheless, the complex genotype–phenotype correlations in XLRP—particularly the extensive phenotypic variability among female carriers—continue to pose considerable challenges in clinical management and genetic counseling (De Silva et al., 2021).

Despite the well-established pathogenic significance of *RPGR*, its precise molecular pathogenic mechanisms remain incompletely elucidated. Most existing studies are largely dependent on mouse models; however, due to substantial interspecies differences in the sequence and function of the RPGR protein, mouse models are unable to fully recapitulate the pathological alterations of the human retina, which has largely hindered the translation of basic research findings into clinical applications (Cehajic-Kapetanovic et al., 2020).

Given the limitations of the aforementioned animal models, recent advances in human induced pluripotent stem cell (hiPSC) technology have offered a new solution. Retinal organoids differentiated from hiPSCs can faithfully recapitulate the key features of the human retina in terms of three-dimensional architecture, cellular composition, and developmental timing (O'Hara-Wright and Gonzalez-Cordero, 2020). Particularly when combined with single-cell RNA sequencing (scRNA-seq) technology, this model enables researchers to systematically dissect transcriptomic perturbations induced by pathogenic mutations and identify the vulnerable cell types at single-cell resolution. This integrated approach paves a new way for directly elucidating the pathological mechanisms of *RPGR* in a human context (Cowan et al., 2020).

This study is based on a unique Chinese pedigree carrying an *RPGR* mutation (p.R826Gfs*8). Beyond confirming its pathogenicity through the standard clinical and genetic examinations, we aim to address a deeper scientific question that extends beyond conventional case reports: how exactly does this mutation disrupt the molecular network of the human retina? To this end, we integrated the detailed data from this pedigree with the previously published scRNA-seq dataset of *RPGR* mutated retinal organoids (SRP535874) (Li et al., 2024). Through this strategy, we sought to systematically dissect the downstream molecular events induced by the mutation from the perspectives of cell-type specificity and developmental dynamics, thereby linking the single mutation site to complex clinical phenotypes via distinct molecular pathways. This not only provides new evidence for understanding the pathogenic mechanism of XLRP but also lays a theoretical foundation for the future development of targeted biomarkers and intervention strategies.

## 2 Materials and methods

### 2.1 Pedigree study and mutation identification

#### 2.1.1 Ethical approval and informed consent

This study adhered to the principles of the Declaration of Helsinki and was approved by the Ethics Review Committee of the People’s Hospital of Ningxia Hui Autonomous Region [Approval ID: (2019) Ethics (Research) No. 397]. Prior to the initiation of the study, the purpose, procedures, and potential risks of the research were fully explained to all the family members participating in this study, and signed written informed consent forms were obtained from each participant.

#### 2.1.2 General information

A Chinese Hui ethnic XLRP pedigree attending Ningxia Eye Hospital was enrolled in this study. The proband reported a history of night blindness since early childhood. The pedigree consisted of six individuals across three generations, including two affected patients. Detailed information regarding the current medical history, past medical history, family history, and parental marriage and reproductive history of all family members was collected through structured interviews and systematically documented, followed by the construction of a pedigree chart. Comprehensive ophthalmic examinations were performed on the proband, his daughter, and his grandson within this pedigree. Anterior segment examinations were conducted using a slit-lamp microscope (Topcon, Japan), and best-corrected visual acuity (BCVA) was measured and recorded using a VT-10 refractometer (Topcon, Japan) and a Snellen visual acuity chart, wide-range color fundus photography (Optos Daytona P200T), high-definition optical coherence tomography (HD-OCT4000, Carl Zeiss Meditec, United States), and fundus autofluorescence (FAF) imaging, perimetry (Humphrey Field Analyzer 750i, Germany), and optical electrophysiological Study examination (ERG, Roland Consult Stasche, Finger GumbHD-14770, Germany).

#### 2.1.3 Genomic DNA extraction and whole-exome sequencing

Peripheral venous blood (5 mL) was collected from the proband and his two sons using EDTA-K2 anticoagulant tubes, thoroughly inverted, and mixed. Genomic DNA was extracted using the QIAamp Blood Mini Kit (QIAGEN, Hilden, Germany). The concentration and purity of the extracted DNA were assessed by ultraviolet spectrophotometry and 1.5% agarose gel electrophoresis. All samples were subsequently stored at −20 °C.

Genomic DNA was extracted from the samples of the subjects to construct a genomic library. Target gene exons along with adjacent splicing regions (approximately 20 bp) and the full length of the mitochondrial genome were captured via probe hybridization and subsequently enriched. Quality control (QC) was performed on the enriched genes, followed by sequencing using a high-throughput sequencer (Illumina HiSeq Xten), achieving an average sequencing depth of 169.99×. The raw sequencing data were first filtered to remove reads that did not meet the QC criteria and then aligned to the human reference genome (hg38 version provided by the University of California, Santa Cruz; https://genome.ucsc.edu/) using BWA software (Burrows–Wheeler aligner). Single nucleotide variants (SNVs) and insertion–deletion variants (InDels) were identified via GATK’s HaplotypeCaller. Copy number variation analysis in the probe-covered regions was conducted using the XHMM and CLAMMS algorithms. A candidate variant was detected in the proband and confirmed in both his two; however, due to the lack of detailed clinical phenotype information for the sons, a complete co-segregation analysis could not be performed. Intrinsic disorder analysis with IUPred3 further confirmed the intrinsically disordered character of the ORF15 protein domain.

### 2.2 Bioinformatics analysis

#### 2.2.1 Acquisition and preprocessing of public datasets

The public scRNA-seq dataset of the *RPGR* mutant retinal organoids (ROs) was utilized in this study. Raw sequencing data (FASTQ files) were downloaded directly from the NCBI Sequence Read Archive (SRA) database under the accession number SRP535874 (Li et al., 2024). This dataset comprises scRNA-seq data of ROs from both the control group and the *RPGR* mutant group across multiple developmental time points (D40, D90, D150, and D200). Raw sequencing data were processed using Cell Ranger software (v7.1.0) for alignment, quantification, and generation of the gene expression count matrix. Alignment was performed against the human reference genome GRCh38 (Ensembl version 104) with the --include-introns parameter enabled to capture reads from both the exonic and intronic regions simultaneously. Stringent QC was implemented for the dataset. Specifically, cells with detected genes ranging from 500 to 6,000 and mitochondrial RNA (mtRNA) content ≤10% were retained, while doublet cells were identified and removed using the DoubletFinder package (v2.0.3) to ensure data integrity. Downstream analyses, including cell clustering, differential expression analysis, and functional enrichment, were conducted based on the preprocessed gene expression matrix. Cell types were annotated using established canonical marker genes for retinal cells.

#### 2.2.2 Data QC, normalization, and integration

Downstream analyses were performed using the Seurat R package (v4.1.1). First, rigorous QC was implemented on the raw gene–cell expression matrix. Cells with fewer than 500 or more than 6,000 detected genes were excluded, and cells with mitochondrial gene content exceeding 10% were also removed. QC thresholds were consistent across all the developmental stages. Doublet detection was performed using DoubletFinder (v2.0.3) with default parameters (including the number of artificial doublets set to 0.25). After stringent QC and doublet removal, a total of 64,323 high-quality single cells were retained for downstream analysis, including 30,338 control cells and 33,985 *RPGR* mutant cells. Gene expression in each cell was normalized using the “NormalizeData” function with the “LogNormalize” method. The normalized data were then scaled using the “ScaleData” function to mitigate technical biases, ensuring that downstream analyses were driven by biological variation rather than technical variations. To integrate different samples and correct for batch effects, the Harmony algorithm (v1.2.0) was applied to align samples based on their origins while preserving the inherent biological heterogeneity. Subsequently, the “FindVariableFeatures” function was used to identify the top 2,000 highly variable genes, which served as the basis for principal component analysis (PCA). Unsupervised cell clustering was performed using the “FindNeighbors” and “FindClusters” functions with the first 16 principal components (which captured the majority of biological variation) and a resolution parameter set to 0.1—optimized to balance cluster distinctiveness and biological relevance. Finally, the “RunUMAP” function was utilized to perform Uniform Manifold Approximation and Projection (UMAP), enabling two-dimensional visualization of the clustered cells to intuitively display cellular distribution and relationships.

#### 2.2.3 Cell-type annotation and differential expression analysis

Cell cluster annotation was completed by integrating the expression of known retinal cell marker genes. Following cell-type annotation, the “FindMarkers” function (default Wilcoxon rank-sum test) was used to perform differential expression analysis on cone photoreceptor and rod photoreceptor subsets separately, aiming to identify the differentially expressed genes (DEGs) in the *RPGR* mutant group compared to those in the control group. The filtering criteria were the absolute value of average log_2_ fold change (|log_2_FC|) > 0.25 and FDR-adjusted *p* < 0.05.

#### 2.2.4 Functional enrichment analysis and PPI network construction

To clarify the potential biological functions of significantly downregulated genes in cone and rod photoreceptors, we performed Gene Ontology (GO) enrichment analysis and Kyoto Encyclopedia of Genes and Genomes (KEGG) pathway analysis. The GO analysis covered three categories, namely, biological processes (BP), molecular functions (MF), and cellular components (CC). The KEGG analysis was used to identify metabolic and signaling pathways that were significantly enriched with DEGs. All the aforementioned analyses were performed using the R package clusterProfiler, with the significance level set at *p* < 0.05. The protein–protein interaction (PPI) network was constructed using the STRING database (v12.0) with a high confidence interaction score >0.7. Hub genes were identified based on the degree centrality using Cytoscape software (v3.10.0).

#### 2.2.5 Pseudotime analysis

To dissect the impact of *RPGR* mutation on the developmental trajectories of cone and rod photoreceptors, pseudotime analysis was performed on the integrated single-cell dataset using the Monocle 3 R package. First, a cell trajectory graph was constructed based on the results of highly variable genes and PCA. Through the reverse graph embedding algorithm, cells were projected onto a branching trajectory structure to simulate their continuous differentiation process. Subsequently, pseudotime values were calculated according to the ordering of cells along the pseudotime axis. Differences in the distribution and developmental paths of cells between the control group and the *RPGR* mutant group on the trajectory were compared.

#### 2.2.6 Data analysis and visualization

All statistical analyses and graph generation were conducted in the R programming environment (v4.1.2). Stacked bar charts for cell type proportions, volcano plots for DEGs, and bubble charts for functional enrichment analysis were plotted using relevant R packages including ggplot2.

## 3 Results

### 3.1 Clinical and genetic profile of a familial case with *RPGR* mutation

**FIGURE 1:**
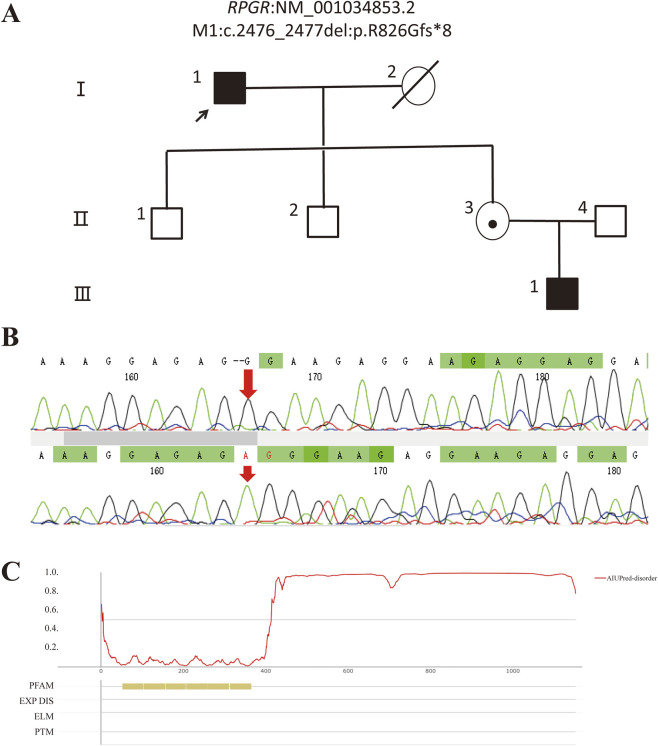
Validation of the variation: **(A)** Pedigree information: Filled black symbols denote affected family members, with the proband indicated by an arrow; II-3 is a carrier. **(B)** Sequencing electropherogram of the RPGR gene. **(C)** IUPred3 prediction of intrinsic disorder in the RPGR protein: The plot shows the AIUPred-disorder score (red line, y-axis, O 1.0) across the full-length RPGR protein sequence (x-axis, amino acid position). Scores >0.5 indicate intrinsically disordered regions (IDRs). The yellow bars (PFAM) mark the N-terminal RCC1-like ordered domain (aa ∼1 - 360). The C-terminal region (aa ∼400 onwards, including the ORF15 domain) exhibits a consistently high disorder score (>0.9), confirming it as a long intrinsically disordered region.

**FIGURE 2:**
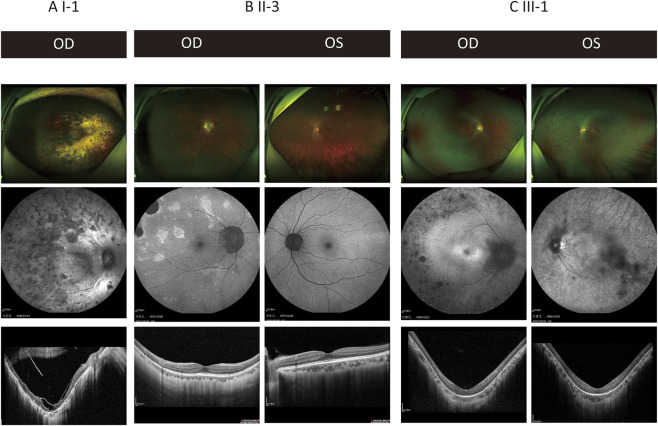
Clinical imaging data of XLRP patients and carrier: **(A)** Proband(I-1): Fundus photo: Attenuated vessels, posterior pole gray atrophy, scattered bone-spicule pigmentation; Fundus autofluorescence: Extensive posterior pole hyperfluorescence with mottled hypofluorescence; Macular OCT: Complete foveal structure destruction, outer nuclear layer (ONL) loss, disrupted ellipsoid zone (IS/OS) and RPE signals, and global retinal disorganization. **(B)** Female carrier (II-3): Fundus photo: Generally normal vessels and macular structure; Fundus autofluorescence: Focal macular hypofluorescence (both eyes) without widespread abnormalities; Macular OCT: Localized IS/OS discontinuity, mild RPE inhomogeneity, and preserved retinal thickness (both eyes). **(C)** Patient (III-1): Fundus photo: Slightly attenuated vessels, peripheral bone-spicule pigmentation; Fundus autofluorescence: Mild macular hyperfluorescence (both eyes); Macular OCT: Preserved foveal contour, localized IS/OS disruption, mild ONL thinning, and intact RPE (both eyes).

**FIGURE 3:**
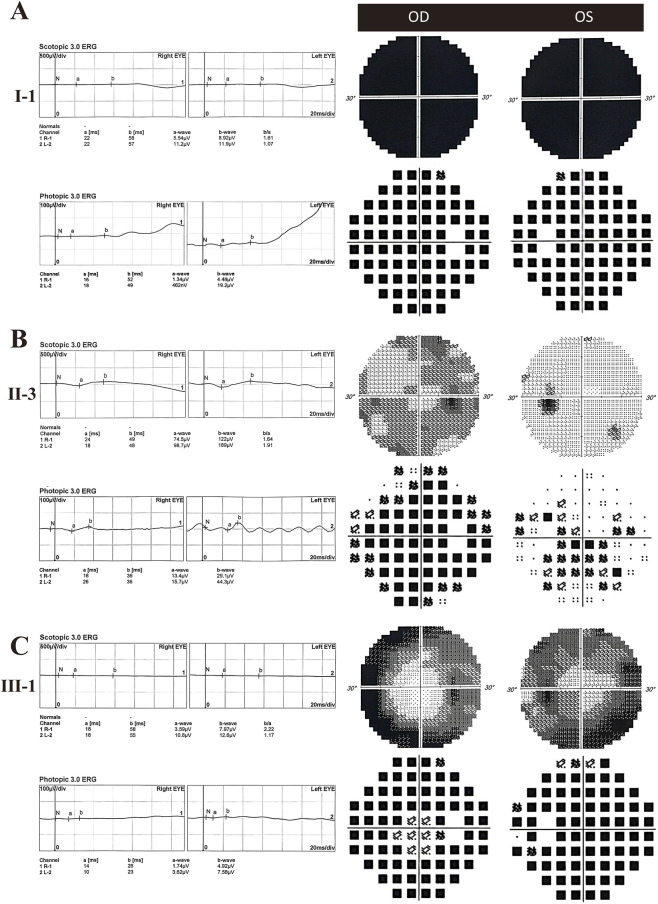
ERG and visual field results of family members: **(A)** Proband (I-1): ERG: a- and b-wave amplitudes are nearly extinguished under both dark-adapted and light-adapted conditions. **(B)** Female carrier (II-3): ERG: Dark-adapted and light-adapted responses are largely preserved, with only mild amplitude reduction; Visual field: Shows mild paracentral scotomas (more severe in the right eye). **(C)** Patient (III-1): ERG shows moderately reduced rod and cone amplitudes; visual field shows diffuse peripheral sensitivity reduction with relatively preserved central vision.

This study enrolled a three-generation Chinese pedigree with XLRP and no history of consanguinity (Figure 1A). The proband (I-1) was an elderly male with a classic severe RP phenotype. He had night blindness since early childhood and progressive visual field constriction. At presentation, his BCVA was no light perception (NLP). Slit-lamp biomicroscopy of the anterior segment revealed an artificial lens in the right eye and a completely opacified lens in the left eye. Fundus examination showed waxy pallor of the optic discs and attenuated retinal vessels; the posterior pole exhibited a pale atrophic retina with extensive bone-spicule pigmentation; macular OCT demonstrated macular atrophy with complete loss of outer retina the outer retinal structures; the signals corresponding to the inner segment/outer segment (IS/OS) junction, retinal pigment epithelium (RPE), and choriocapillaris complex were barely discernible; the foveal contour was effaced; choroidal thinning was observed; FAF revealed a large area of hypoautofluorescence in the posterior pole, with only residual peripheral islands of hyperautofluorescence (Figure 2A). ERG recordings showed non-detectable responses under both scotopic (0.01, 3.0, and 10.0 cd·s/m^2^) and photopic (3.0 cd·s/m^2^) conditions. The a- and b-waves were nearly unidentifiable, and the waveforms under the photopic conditions were extremely flattened. The amplitudes in both the eyes were less than 10% of the lower limit of normal, indicating a near-total loss of function in both the rod and cone systems (Figure 3A).

The proband’s daughter presented with bilateral refractive errors, including high myopia in the right eye. Her BCVA was 0.15 in the right eye (−14.00 DS/−3.00 DC × 35°) and 0.6 in the left eye (−4.75 DS/−3.50 DC × 155°). Slit-lamp biomicroscopy revealed mild lens opacities in both eyes. Fundus photography demonstrated attenuated retinal vessels without bone-spicule pigmentation, and OCT showed a preserved foveal contour, though with discontinuous signals in the ellipsoid zone and external limiting membrane; there was mild reduction in the sub-foveal retinal thickness, accompanied by thinning of the RPE and moderate choroidal thinning; the inner retinal layers remained intact (Figure 2B). Visual field testing revealed paracentral scotomas and ring-shaped defects, which were more pronounced in the right eye, and ERG indicated mild-to-moderate reductions in the amplitudes of both rod and cone responses, with normal implicit times and non-extinguished waveforms (Figure 3B). Collectively, these findings showed a typical intermediate phenotype characteristic of a female carrier of XLRP, wherein structural damage primarily involves the photoreceptor–RPE interface in the macular region with relative sparing of the peripheral retina. The ERG profile demonstrates bilateral mild-to-moderate dysfunction of both the rod and cone systems. The asymmetric visual field loss was more severe in the right eye, consistent with the asymmetric phenotype in female carriers of XLRP.

The proband’s grandson also had bilateral refractive errors, with a BCVA of 0.25 in the right eye (−4.25 DS/−0.75 DC × 170°) and 0.3 in the left eye (−4.00 DS/−0.75 DC × 10°). No significant abnormalities were noted on the anterior segment slit-lamp examination. Fundus photography showed attenuated retinal vessels with peripheral bone-spicule pigment deposits; the foveal reflex was present, and FAF displayed patchy hypoautofluorescence in the posterior pole; OCT imaging confirmed the preservation of the foveal contour but revealed disruptions in the IS/OS junction and external limiting membrane (ELM), and there was mild reduction in retinal thickness, along with thinning of the RPE and mild choroidal thinning, while the inner retinal architecture was normal (Figure 2C). Visual field testing indicated a ring-shaped depression of sensitivity in both eyes. ERG recordings showed mild-to-moderate amplitude reductions in both cone and rod responses, with normal implicit times and non-extinguished waveforms (Figure 3C). These observations are consistent with early-stage changes in an XLRP-affected male. Intraocular pressure was within the normal range for all the examined individuals. A summary of the ophthalmic examinations for the key pedigree members is provided in Table 1.

**TABLE 1:** Ophthalmic examinations of XLRP pedigree members.

Subject	Sex	Age	Eye	Refraction	BCVA	IOP (mmHg)
I-1 II-3 III-1	M F M	79 50 28	OD OS OD OS OD OS	pl pl −14.00DS/-3.00DCx35° −4.75DS/-3.50DCx155° −4.25DS/-0.75DCx170° −4.00DS/-0.75DCx10°	NLP NLP 0.15 0.6 0.25 0.3	13 11 14 15 14 13

F, female; M, male; OD, oculus dexter; OS, oculus sinister; BCVA, best-corrected visual acuity.

### 3.2 Genetic testing results

Whole-exome sequencing identified a hemizygous frameshift deletion variant in the *RPGR* gene of the proband, namely, c.2476_2477del (p.R826Gfs*8). Sanger sequencing confirmed that this variant was not detected in his son at the corresponding locus (Figure 1B). This frameshift deletion causes a glycine substitution at position 826 and introduces a premature termination codon, leading to a truncated *RPGR* protein. The mutation is located within the intrinsically disordered ORF15 domain, which is critical for *RPGR* function in photoreceptor ciliary transport (Megaw et al., 2015) (Figure 1C). According to the ClinGen X-linked Inherited Retinal Disease Expert Panel Specifications and the ACMG/AMP RPGR variant interpretation guidelines (v1.0.0), this variant has been reported previously in ClinVar (RCV004813740) and classified as pathogenic in prior studies. However, complete co-segregation analysis could not be performed in the present pedigree due to the limited clinical phenotypic information from the available family members. According to the ACMG criteria, this variant is, therefore, classified as likely pathogenic (LP) (PVS1, PM2_Supporting).

While the clinical significance of this mutation is well-established, the molecular mechanisms underlying its photoreceptor-specific toxicity remain incompletely understood. Given the difficulty in obtaining patient retinal tissue for molecular biological analysis, we utilized a published single-cell transcriptomic dataset of retinal organoids harboring an RPGR exon 14–15 deletion to analyze its pathogenic mechanisms at the cellular and molecular level. Although the c.2476_2477del mutation identified in this study lies within the genomic region encompassed by the exon 14–15 deletion in the public dataset, the two mutations produce qualitatively different truncated proteins, namely, the present patient’s mutation retains the RCC1-like domain but loses the C-terminal glutamylation substrate region, whereas the exon 14–15 deletion eliminates the entire RCC1-like domain. The present patient’s mutation retains the RCC1-like domain but loses the C-terminal glutamylation substrate region, whereas the exon 14–15 deletion eliminates the entire RCC1-like domain.

### 3.3 Single-cell transcriptomics reveals retinal developmental abnormalities and molecular dysfunction induced by *RPGR* mutation

**FIGURE 4:**
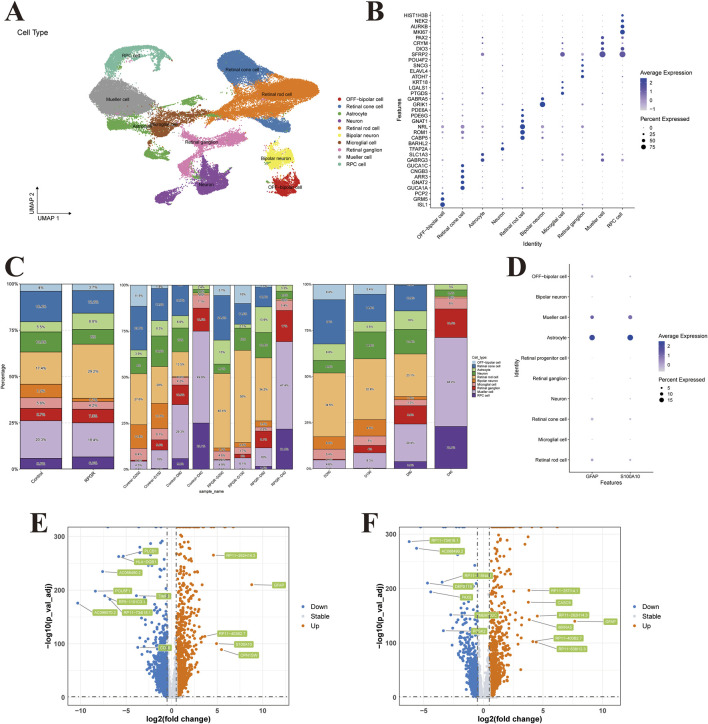
Cell type atlas of control and RPGR mutant retinal organoids. **(A)** UMAP plot showing the 10 annotated retinal cell types. **(B)** Dot plot displaying canonical marker genes for the 10 major cell types identified from the scRNA-seq data. **(C)** Stacked bar plots of cell proportions, comparing the distribution of cell type proportions across different groups, deve -lopmental stages, and four stages of control vs. RPGR groups in retinal organoid samples. **(D)** Dot plot showing expressio -n patterns of GFAP and S100A10 across major retinal cell types. **(E)** Volcano plots of DEGs between control and RPGR -mutant groups in cone photoreceptors. **(F)** Volcano plots of DEGs between control and RPGR-mutant groups in rod pho -toreceptors.

To analyze the impact of the *RPGR* mutation at the cellular and molecular levels, we performed an in-depth bioinformatics analysis on a published scRNA-seq dataset derived from *RPGR* mutant retinal organoids. This dataset encompasses transcriptomic profiles from both the control and *RPGR* mutant groups across four developmental time-points. We performed unbiased clustering and UMAP dimensionality reduction on single-cell transcriptomic profiles, yielding 10 distinct cell clusters. Using canonical retinal marker genes, we annotated these clusters and validated their expression specificity by dot plot analysis (Figure 4B). The annotated UMAP plot displays 10 major retinal cell types, including rod photoreceptors, cone photoreceptors, OFF-bipolar cells, bipolar neurons, retinal ganglion cells, Müller glia, astrocytes, microglia, and retinal progenitor cells (RPCs) (Figure 4A). The normal developmental trajectory of retinal organoids from the early to late stages was delineated based on the quantitative analysis of cell proportion stacking plots. Through direct comparison of the cellular composition between the control and *RPGR* mutant groups at matched time-points, we observed that during the late developmental stages (D150 and D200), the *RPGR* mutant organoids showed an increasing trend in the transcriptional activity or relative cellular abundance of photoreceptor cells, such as cones and rods (Figure 4C). Concurrently, macular OCT imaging from our *RPGR* pedigree patient revealed typical progressive atrophy in the retinal outer layers (Figure 2). Together, these findings indicate a potential “transcriptional abundance increase–functional structure loss” paradox, prompting the hypothesis that *RPGR* loss-of-function mutations may drive excessive yet disordered production of photoreceptor precursors, which fail to mature into functional units. However, as the scRNA-seq data derive from a different allele (exon 14–15 deletion), this hypothesis requires direct testing in a model carrying the specific c.2476_2477del mutation.

**FIGURE 5:**
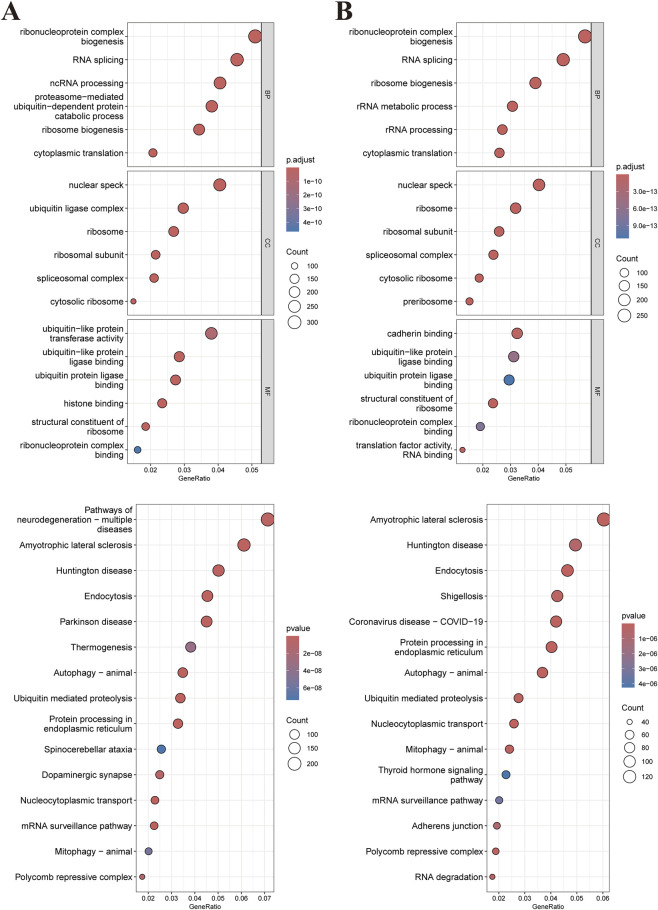
Functional enrichment analysis of DEGs in cone and rod photoreceptors. **(A)** GO and KEGG enrichment plots for DEGs in cone photoreceptors. **(B)** GO and KEGG enrichment plots for DEGs in rod photoreceptors. Dot size indicates the number of enriched genes; color represents the P-value.

To delineate the molecular alterations in the affected cells, we focused on differential gene expression analysis within rod and cone photoreceptors. Volcano plots revealed that the *RPGR* mutation induced extensive transcriptional dysregulation in both cell types. Within the photoreceptor clusters, we identified numerous differentially expressed genes involved in phototransduction and ciliary transport. We also detected upregulated genes associated with glial activation and cellular stress, including *GFAP* and *S100A10* (Figures 4E,F). Dot plot analysis verified that *GFAP* was specifically expressed in Müller glia, whereas *S100A10* was mainly distributed in glial cells and associated with glial activation and cellular stress (Figure 4D). Subsequent GO and KEGG pathway enrichment analyses were performed on these DEGs. KEGG analysis indicated significant enrichment for “neurodegenerative disease pathways” (e.g., amyotrophic lateral sclerosis and Huntington’s disease) in both rods and cones, with both also implicating the “ubiquitin-mediated proteolysis” pathway. GO enrichment analysis showed that rod photoreceptors were significantly enriched for biological processes such as “ribosome biogenesis,” “rRNA metabolic process,” and “RNA splicing.” Cone photoreceptors were additionally enriched for the “ubiquitin-dependent protein catabolic process” (Figure 5A,B). These results collectively indicate that the *RPGR* mutation severely disrupts protein synthesis and RNA metabolism, potentially leading to the accumulation of misfolded proteins and a comprehensive collapse of cellular function.

**FIGURE 6:**
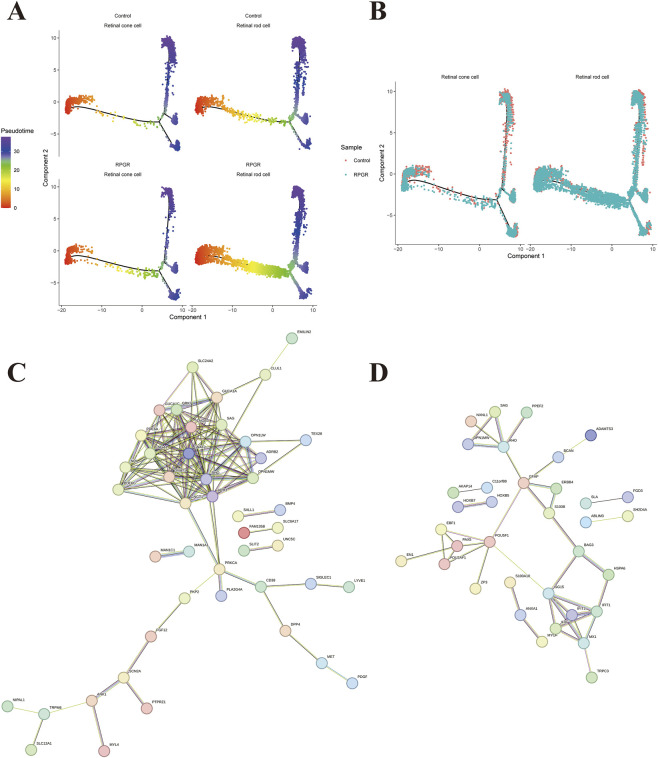
Pseudotime trajectory and PPI network analysis of photoreceptors. **(A)** Pseudotime trajectory of photoreceptors: Pseudotime trajectory distribution of cone/rod cells in control and RPGR mutant groups (color indicates pseudotime). Control group cells cover a complete maturation trajectory, while mutant group cells are concentrated in the intermediate differentiation stage. **(B)** PPI networks of DEGs: PPI network of DEGs in cone cells (left) and rod cells (right).

Pseudotime trajectory analysis revealed that the *RPGR* mutation significantly disrupted the differentiation trajectories of both cone and rod photoreceptors. In contrast to control cells, which showed a continuous and orderly distribution along the pseudotime axis (0→30) from the early precursor states to terminal maturation, photoreceptor cells in the *RPGR* mutant group were predominantly enriched in the intermediate pseudotime range. Notably, cells reaching the high pseudotime stage, representing the terminal mature states, were nearly absent (Figures 6A,B). These results indicate that the *RPGR* mutation may cause a blockade in the photoreceptor differentiation process at an intermediate stage, preventing cells from progressing normally into the functionally mature terminal state. PPI network analysis indicates that the *RPGR* mutation disrupts the phototransduction complex in rod photoreceptors. Concurrently, a distinct stress-response module centered on glial activation was markedly activated, with *GFAP* and *S100A10* as key nodes involved in glial and inflammatory responses. These changes indicate a coordinated network that links photoreceptor dysfunction and glial activation in *RPGR* mutant retinal organoids (Figures 6C,D). Violin plot visualization confirmed that *GFAP* was specifically expressed in Müller glia rather than photoreceptors, and its appearance in the DEG list was attributed to minor cluster contamination. Notably, *GFAP* and *S100A10* upregulation was derived from glial cells rather than photoreceptors, confirming reactive gliosis secondary to photoreceptor damage, with no evidence of aberrant expression within the photoreceptor clusters. The characteristics of this network may offer potential mechanistic clues that are relevant to XLRP progression. However, these observations are derived from a different *RPGR* allele and should be considered hypothesis-generating until validated in models carrying the specific c.2476_2477del variant.

## 4 Discussion

This study integrated clinical data from an XLRP pedigree carrying an *RPGR* mutation (c.2476_2477del) with a public single-cell transcriptomic dataset, deciphering the molecular and cellular mechanisms underlying retinal degeneration induced by this mutation at single-cell resolution. It not only reports a likely pathogenic variant but, more importantly, establishes a multi-tiered mechanistic framework linking the genotype to clinical phenotype.

First, the *RPGR* c.2476_2477del (p.R826Gfs*8) mutation identified in a large pedigree is located within the critical ORF15 region of the gene. This mutation occurs within the intrinsically disordered ORF15 domain and produces a truncated loss-of-function protein. This is consistent with the established pathogenic mechanism of *RPGR*-associated XLRP (Tee et al., 2016; Megaw et al., 2015). This variant has been reported previously in ClinVar (RCV004813740), and our findings further support its pathogenic potential. This discovery expands the mutational spectrum of the *RPGR* gene and provides a critical molecular foundation for genetic counseling and potential future gene therapy for this family (Sladen et al., 2024).

Given the significant ethical and technical challenges of directly studying molecular changes in human retinal tissue, the integration of human iPSC-derived retinal organoids with single-cell transcriptomic technology has become a powerful tool for understanding human-specific retinal development and disease mechanisms (Sridhar et al., 2020; Collin et al., 2019). This study innovatively utilized a public database encompassing a large *RPGR* deletion (exons 14–15), a region that includes the c.2476_2477del site we identified. This strategy allowed us to move beyond a mere case report and delve into its downstream biological effects.

Before interpreting the mechanistic findings below, it is important to recall the key distinction between our patient’s mutation and the public dataset, as detailed in the Results section (Section 3.2). The public dataset models an exon 14–15 deletion that is qualitatively different from our patient’s c.2476_2477del frameshift mutation. Consequently, the transcriptomic alterations and mechanistic inferences described below, derived from the exon 14–15 deletion model, should be regarded as hypothesis-generating observations that require validation in a model specifically carrying the c.2476_2477del allele.

In this study, *RPGR* mutant organoids showed aberrantly elevated photoreceptor transcriptional activity at late developmental stages, representing a conspicuous paradox with the structural loss of the functional photoreceptor layer observed in patient OCT images. This “increased transcriptional abundance coupled with the loss of structural function” phenotype is consistent with the previously reported pathological features of *RPGR* mutations, including outer nuclear layer thinning and opsin mislocalization (Sladen et al., 2024; Deng et al., 2018; Chahine Karam et al., 2022). We hypothesize that this apparent contradiction may stem from an *RPGR* mutation-induced aberrant state characterized by “excessive yet disorganized and functionally deficient photoreceptor precursor production.” Although these cells exhibit a mature transcriptional profile, they likely harbor severe defects in protein synthesis, subcellular localization, and ultimate polarity establishment, preventing their proper integration into a functionally competent outer nuclear layer structure. This hypothesis offers a novel perspective for understanding the cellular mechanism of *RPGR*-associated retinal degeneration, namely, disease progression may not only be caused by cell death but is also closely linked to aberrant cell fate decisions and subsequent failure in functional maturation. In summary, the integration of our single-cell transcriptomic data with patient clinical imaging indicates potential cellular phenotypes driven by the *RPGR* loss-of-function mutation in the context of the exon 14–15 deletion model. Whether these findings directly apply to the c.2476_2477del mutation remains to be determined.

To elucidate the molecular drivers of disrupted cell fate, this study constructed a PPI network based on DEGs in rod and cone photoreceptors from the *RPGR* mutant group. Integrated with GO and KEGG pathway enrichment analyses, this approach further indicated two core biological processes that may be affected, namely, “ciliary function/intracellular transport” and “phototransduction.” This finding is consistent with the established biological role of the RPGR protein, which localizes to the connecting cilium of the retinal photoreceptors and mediates the transport of key functional proteins (Awadh Hashe et al., 2023; Wright et al., 2011).

Single-cell transcriptomic analysis of DEGs indicated that the *RPGR* loss-of-function mutation was associated not only with downregulation of core phototransduction pathway genes (e.g., RHO, GNGT1, and SAG) but also with dysregulated expression of genes involved in ciliary transport (e.g., certain IFT family and BBS-related genes). We further constructed a PPI network and integrated GO functional and KEGG pathway enrichment analyses. These integrated analyses indicated significant rewiring of the interaction network among these DEGs, characterized by weakened connections between the phototransduction and ciliary transport modules, alongside a relative increase in connectivity within a glial activation and cellular stress module centered on genes such as *S100A10*. Violin plot visualization confirmed that *GFAP* was specifically expressed in Müller glia rather than photoreceptors, and its presence in the DEG list was attributed to minor cluster contamination. Collectively, these data imply that the pathological process triggered by the *RPGR* loss-of-function mutation may not represent a linear failure of a single pathway. Instead, it may initiate a cascade reaction, beginning with a functional defect in a critical cellular structure (the connecting cilium), leading to the decoupling of core physiological modules (ciliary transport and phototransduction), and potentially culminating in a disruption of cellular homeostasis.

First, the RPGR protein is definitively localized to the connecting cilium, which is a critical gateway that links the inner and outer segments of photoreceptors and regulates protein trafficking to the outer segment (Hong et al., 2003). Second, *RPGR* interacts via its RCC1-like domain with proteins such as PDEδ, forming a complex “*RPGR* interactome” that directly governs transport mechanisms involving RAB8 and the directional trafficking of key functional proteins including opsins (Wätzlich et al., 2013; Murga-Zamalloa et al., 2010). Loss of *RPGR* function leads to the mislocalization of proteins such as opsins and disrupts the transport of phototransduction proteins, constituting the core pathological basis for photoreceptor cell death and vision loss (Hong et al., 2000; Zhang et al., 2002; Thompson et al., 2012). The present study, at the single-cell transcriptomic level, is consistent with the notion that *RPGR* loss-of-function disrupts this “gating” and transport function of the connecting cilium, thereby cascading into interference with the downstream phototransduction process.

Specifically, the underlying logic manifests as a multi-tiered progressive model, namely, the loss of *RPGR* function primarily disrupts the efficiency of the ciliary transport system, leading to the mislocalization and faulty trafficking of key molecules such as opsins (Hong et al., 2003). The rewiring of the PPI network may then signify a state of compensatory failure or pathological shift within the cell’s homeostatic regulatory network as it contends with this series of internal crises. Within this context, *GFAP*—a classic marker of Müller glial activation—was significantly upregulated at the whole dataset level, which likely reflects glial reactivity in response to photoreceptor degeneration rather than aberrant expression in the photoreceptors themselves. Concurrently, the enhanced association of *S100A10* may point toward dysregulated calcium signaling or the establishment of a chronic inflammatory microenvironment. Consequently, the single-cell transcriptomic and interactome data from this study indicate a potential mechanism that requires further analysis, namely, the *RPGR* loss-of-function mutation may disrupt the structural and functional coupling of the “ciliary transport–phototransduction axis,” thereby potentially pushing cells into a state of sustained stress and homeostatic imbalance.

This provides support for a systematic molecular framework for understanding *RPGR*-associated photoreceptor-specific degeneration. This molecular cascade model offers a tentative explanation for the multi-layered phenotypes observed in both the organoids and patients, namely, early dysfunction in ciliary transport and phototransduction may correspond to the initial loss of rod cell function and onset of night blindness in patients (Huang et al., 2012), whereas the aberrant activation of stress modules and potential cellular identity disruption may be closely linked to the massive apoptosis of photoreceptors, gliosis, and the progressive and irreversible loss of visual function that is characteristic of mid-to-late-stage disease. Based on this model, future therapeutic strategies may need to extend beyond traditional single-target approaches such as gene replacement or neuroprotection and instead consider multi-target synergistic interventions aimed at various steps of this multi-link cascade process.

Consequently, based on the exon 14–15 deletion model, this study proposes a hypothetical pathogenic model for XLRP, namely, the *RPGR* loss-of-function mutation may perturb the protein interaction network mediated by the DEGs, simultaneously severing both the “vital lifeline” (ciliary transport function) and the “functional core” (phototransduction pathway) of the photoreceptors. This may ultimately trigger photoreceptor differentiation arrest, functional collapse, and progressive cell death. This model is consistent with the “interrupted photoreceptor maturation trajectory” observed in the public dataset and the clinical phenotypes seen in our familial cases—namely, “progressive thinning of the outer nuclear layer” and “progressive loss of ERG function.”

A key finding of this study is that in the *RPGR* mutation model, the number of cells expressing photoreceptor markers (e.g., L/M Opsin and NRL) did not decrease and was even increased, yet the proportion of fully functional and mature cells declined. This “quantity–quality dissociation” phenomenon aligns with findings from other studies, namely, the presence of “ectopic and dysfunctional photoreceptors” in NR2E3-mutant organoids (Mullin et al., 2024) and the “abnormally increased but functionally impaired cone proportion” in RP2-knockout organoids (Lane et al., 2020). This consistency indicates that certain genetic mutations can cause a decoupling between the “expression of lineage markers” and the “achievement of functional maturity” in photoreceptors. Consequently, it implies that the evaluation of future therapeutic strategies must consider both the “maintenance of cell numbers” and “restoration of function,” thereby introducing a new dimension for selecting the therapeutic targets in XLRP.

Clinical translation and therapeutic implications. Given that AAV-mediated *RPGR* gene therapy is currently under active clinical development for XLRP (Michaelides et al., 2024), the c.2476_2477del variant identified in this study holds important translational implications. This variant lies within the *RPGR* ORF15 region, which is the canonical target of current AAV-based *RPGR* gene replacement therapies. Since such approaches deliver a full-length functional *RPGR* coding sequence regardless of the specific mutation within ORF15, patients carrying this frameshift deletion are potentially eligible for existing AAV-mediated *RPGR* therapeutic regimens (Awadh Hashe et al., 2023). This supports the clinical relevance of our genetic findings for future personalized therapy and genetic counseling.

Our study also has certain limitations. First, the public database utilizes a large-fragment deletion model, whereas we analyzed a specific small-fragment deletion mutation. Although the two overlap in the genomic region and are both predicted to be loss-of-function variants, their molecular phenotypes may differ in subtle aspects. Second, while retinal organoids recapitulate key aspects of human retinal development, they lack several critical components of the native retinal microenvironment, including blood vessels (vasculature) and immune cells (e.g., microglia and infiltrating immune cells). The absence of these components may influence the interpretation of our single-cell transcriptomic findings in two ways: (1) the observed stress-responses and glial activation patterns may be attenuated or qualitatively different compared to those in the *in vivo* retina, where vascular dysfunction and immune infiltration contribute to disease progression; (2) late-stage degenerative phenotypes, such as photoreceptor cell death and tissue remodeling, may be less pronounced in organoids than in patients. Consequently, the transcriptomic alterations reported here likely represent cell-autonomous effects of *RPGR* loss-of-function mutation, whereas non-cell-autonomous contributions from the vascular and immune systems remain unexplored. Future research could involve co-culture systems incorporating retinal pigment epithelium, vascular endothelial cells, and immune cells or the use of animal models to complement the organoid findings.

In summary, based on a different *RPGR* allele (exon 14–15 deletion), our study delineates a hypothesis-generating pathogenic roadmap that requires validation in models carrying the patient-specific c.2476_2477del mutation, progressing through the disruption of protein interaction networks, leading to photoreceptor maturation arrest and cell death, and ultimately culminating in clinical retinal degeneration. This work not only confirms the significant utility of public databases in aiding the interpretation of clinically rare mutations but also lays a theoretical foundation for the development of targeted therapies against this specific variant.

## Data Availability

The public scRNA-seq dataset analyzed in this study is publicly available in the NCBI Sequence Read Archive (SRA) under the accession number SRP535874. Clinical and genetic data generated in this study are included within the article and supporting figures. Custom code used for bioinformatic analyses is available from the corresponding author upon reasonable request.

## References

[B1] Awadh Hashem S. Georgiou M. Ali R. R. Michaelides M. (2023). *RPGR*-related retinopathy: clinical features, molecular genetics, and gene replacement therapy. Cold Spring Harb. Perspect. Med. 13, a041280. 10.1101/cshperspect.a041280 37188525 PMC10626266

[B2] Cehajic-Kapetanovic J. Xue K. Martinez-Fernandez de la Camara C. Nanda A. Davies A. Wood L. J. (2020). Initial results from a first-in-human gene therapy trial on X-linked retinitis pigmentosa caused by mutations in *RPGR* . Nat. Med. 26 (3), 354–359. 10.1038/s41591-020-0763-1 32094925 PMC7104347

[B3] Chahine Karam F. Loi T. H. Ma A. Nash B. M. Grigg J. R. Parekh D. (2022). Human iPSC-derived retinal organoids and retinal pigment epithelium for novel intronic *RPGR* variant assessment for therapy suitability. J. Pers. Med. 12 (3), 502. 10.3390/jpm12030502 35330501 PMC8951517

[B4] Collin J. Queen R. Zerti D. Dorgau B. Hussain R. Coxhead J. (2019). Deconstructing retinal organoids: single cell RNA-seq reveals the cellular components of human pluripotent stem cell-derived retina. Stem Cells 37 (5), 593–598. 10.1002/stem.2963 30548510 PMC6519347

[B5] Cowan C. S. Renner M. De Gennaro M. Gross-Scherf B. Goldblum D. Hou Y. (2020). Cell types of the human retina and its organoids at single-cell resolution. Cell 182 (6), 1623–1640.e34. 10.1016/j.cell.2020.08.013 32946783 PMC7505495

[B6] De Silva S. R. Arno G. Robson A. G. Fakin A. Pontikos N. Mohamed M. D. (2021). The X-linked retinopathies: physiological insights, pathogenic mechanisms, phenotypic features and novel therapies. Prog. Retin. Eye Res. 82, 100898. 10.1016/j.preteyeres.2020.100898 32860923

[B7] Deng W. L. Gao M. L. Lei X. L. Lv J. N. Zhao H. He K. W. (2018). Gene correction reverses ciliopathy and photoreceptor loss in iPSC-derived retinal organoids from retinitis pigmentosa patients. Stem Cell Rep. 10 (4), 1267–1281. 10.1016/j.stemcr.2018.02.003 29526738 PMC5998840

[B8] Hong D. H. Pawlyk B. S. Shang J. Sandberg M. A. Berson E. L. Li T. (2000). A retinitis pigmentosa GTPase regulator (*RPGR*)-deficient mouse model for X-linked retinitis pigmentosa (RP3). Proc. Natl. Acad. Sci. U.S.A. 97 (7), 3649–3654. 10.1073/pnas.97.7.3649 10725384 PMC16294

[B9] Hong D. H. Pawlyk B. Sokolov M. Strissel K. J. Yang J. Tulloch B. (2003). *RPGR* isoforms in photoreceptor connecting cilia and the transitional zone of motile cilia. Invest. Ophthalmol. Vis. Sci. 44 (6), 2413–2421. 10.1167/iovs.02-1206 12766038

[B10] Huang W. C. Wright A. F. Roman A. J. Cideciyan A. V. Manson F. D. Gewaily D. Y. (2012). *RPGR*-associated retinal degeneration in human X-linked RP and a murine model. Invest. Ophthalmol. Vis. Sci. 53 (9), 5594–5608. 10.1167/iovs.12-10070 22807293 PMC3422104

[B11] Karuntu J. S. Almushattat H. Nguyen X. T. Plomp A. S. Wanders R. J. A. Hoyng C. B. (2025). Syndromic retinitis pigmentosa. Prog. Retin. Eye Res. 107, 101324. 10.1016/j.preteyeres.2024.101324 39733931

[B12] Lane A. Jovanovic K. Shortall C. Ottaviani D. Panes A. B. Schwarz N. (2020). Modeling and rescue of *RP2* retinitis pigmentosa using iPSC-derived retinal organoids. Stem Cell Rep. 15 (1), 67–79. 10.1016/j.stemcr.2020.05.007 32531192 PMC7363745

[B13] Li T. Ma Y. Cheng Y. Zhao Y. Qiu Z. Liu H. (2024). Single-cell transcriptomic dataset of *RPGR*-associated retinitis pigmentosa patient-derived retinal organoids. Sci. Data 11 (1), 1285. 10.1038/s41597-024-04124-z 39592612 PMC11599861

[B14] Megaw R. D. Soares D. C. Wright A. F. (2015). *RPGR*: its role in photoreceptor physiology, human disease, and future therapies. Exp. Eye Res. 138, 32–41. 10.1016/j.exer.2015.06.007 26093275 PMC4553903

[B15] Michaelides M. Besirli C. G. Yang Y. DE Guimaraes T. A. C. Wong S. C. Huckfeldt R. M. (2024). Phase 1/2 AAV5-hRKp. *RPGR* (Botaretigene sparoparvovec) gene therapy: safety and efficacy in *RPGR*-associated X-Linked retinitis pigmentosa. Am. J. Ophthalmol. 267, 122–134. 10.1016/j.ajo.2024.05.034 38871269

[B16] Mullin N. K. Bohrer L. R. Voigt A. P. Lozano L. P. Wright A. T. Bonilha V. L. (2024). *NR2E3* loss disrupts photoreceptor cell maturation and fate in human organoid models of retinal development. J. Clin. Invest. 134 (11), e173892. 10.1172/JCI173892 38652563 PMC11142732

[B17] Murga-Zamalloa C. A. Atkins S. J. Peranen J. Swaroop A. Khanna H. (2010). Interaction of retinitis pigmentosa GTPase regulator (*RPGR*) with RAB8A GTPase: implications for cilia dysfunction and photoreceptor degeneration. Hum. Mol. Genet. 19 (18), 3591–3598. 10.1093/hmg/ddq275 20631154 PMC2928130

[B18] O'Hara-Wright M. Gonzalez-Cordero A. (2020). Retinal organoids: a window into human retinal development. Development 147 (24), dev189746. 10.1242/dev.189746 33361444 PMC7774906

[B19] Sladen P. E. Naeem A. Adefila-Ideozu T. Vermeule T. Busson S. L. Michaelides M. (2024). AAV-*RPGR* gene therapy rescues opsin mislocalisation in a human retinal organoid model of *RPGR*-associated X-linked retinitis pigmentosa. Int. J. Mol. Sci. 25 (3), 1839. 10.3390/ijms25031839 38339118 PMC10855600

[B20] Sridhar A. Hoshino A. Finkbeiner C. R. Chitsazan A. Dai L. Haugan A. K. (2020). Single-cell transcriptomic comparison of human fetal retina, hPSC-derived retinal organoids, and long-term retinal cultures. Cell Rep. 30 (5), 1644–1659.e4. 10.1016/j.celrep.2020.01.007 32023475 PMC7901645

[B21] Tee J. J. Smith A. J. Hardcastle A. J. Michaelides M. (2016). *RPGR*-associated retinopathy: clinical features, molecular genetics, animal models and therapeutic options. Br. J. Ophthalmol. 100 (8), 1022–1027. 10.1136/bjophthalmol-2015-307698 26843488

[B22] Thompson D. A. Khan N. W. Othman M. I. Chang B. Jia L. Grahek G. (2012). Rd9 is a naturally occurring mouse model of a common form of retinitis pigmentosa caused by mutations in *RPGR*-ORF15. PLoS One 7 (5), e35865. 10.1371/journal.pone.0035865 22563472 PMC3341386

[B23] Wätzlich D. Vetter I. Gotthardt K. Miertzschke M. Chen Y. X. Wittinghofer A. (2013). The interplay between *RPGR*, PDEδ and Arl2/3 regulate the ciliary targeting of farnesylated cargo. EMBO Rep. 14 (5), 465–472. 10.1038/embor.2013.37 23559067 PMC3642377

[B24] Wright R. N. Hong D. H. Perkins B. (2011). Misexpression of the constitutive *Rpgr*(ex1-19) variant leads to severe photoreceptor degeneration. Invest. Ophthalmol. Vis. Sci. 52 (8), 5189–5201. 10.1167/iovs.11-7470 21546531 PMC3176051

[B25] Zhang Q. Acland G. M. Wu W. X. Johnson J. L. Pearce-Kelling S. Tulloch B. (2002). Different *RPGR* exon ORF15 mutations in canids provide insights into photoreceptor cell degeneration. Hum. Mol. Genet. 11 (9), 993–1003. 10.1093/hmg/11.9.993 11978759

